# NAFLD in the 21st Century: Current Knowledge Regarding Its Pathogenesis, Diagnosis and Therapeutics

**DOI:** 10.3390/biomedicines12040826

**Published:** 2024-04-09

**Authors:** Dimitris Kounatidis, Natalia G. Vallianou, Eleni Geladari, Maria Paraskevi Panoilia, Anna Daskou, Theodora Stratigou, Irene Karampela, Dimitrios Tsilingiris, Maria Dalamaga

**Affiliations:** 1 Department of Internal Medicine, Hippokration General Hospital, 114 Vassilissis Sofias str, 11527 Athens, Greece; dimitriskounatidis82@outlook.com; 2 First Department of Internal Medicine, Sismanogleio General Hospital, 1 Sismanogliou str, 15126 Athens, Greece; 3 Department of Internal Medicine, Evangelismos General Hospital, 45–47Ipsilantou str, 10676 Athens, Greece; 4 Department of Endocrinology and Metabolism, Evangelismos General Hospital, 45–47Ipsilantou str, 10676 Athens, Greece; 5 2nd Department of Critical Care, Medical School, Attikon General University Hospital, University of Athens, 1 Rimini str., 12461 Athens, Greece; 6 First Department of Internal Medicine, University Hospital of Alexandroupolis, Demokritus University of Thrace, 68100 Alexandroupoli, Greece; 7 Department of Biological Chemistry, Medical School, National and Kapodistrian University of Athens, 75 Mikras Asias str., 11527 Athens, Greece; madalamaga@med.uoa.gr

**Keywords:** GLP-1 analogues, NAFLD, tirzepatide, probiotics, prebiotics, pegozafermin, resmetiron

## Abstract

Non-alcoholic fatty liver disease (NAFLD) is a major public health issue worldwide. It is the most common liver disease in Western countries, andits global prevalence is estimated to be up to 35%. However, its diagnosis may be elusive, because liver biopsy is relatively rarely performed and usually only in advanced stages of the disease. Therefore, several non-invasive scores may be applied to more easily diagnose and monitor NAFLD. In this review, we discuss the various biomarkers and imaging scores that could be useful in diagnosing and managing NAFLD. Despite the fact that general measures, such as abstinence from alcohol and modulation of other cardiovascular disease risk factors, should be applied, the mainstay of prevention and management is weight loss. Bariatric surgery may be suggested as a means to confront NAFLD. In addition, pharmacological treatment with GLP-1 analogues or the GIP agonist tirzepatide may be advisable. In this review, we focus on the utility of GLP-1 analogues and GIP agonists in lowering body weight, their pharmaceutical potential, and their safety profile, as already evidenced inanimal and human studies. We also elaborate on other options, such as the use of vitamin E, probiotics, especially next-generation probiotics, and prebiotics in this context. Finally, we explore future perspectives regarding the administration of GLP-1 analogues, GIP agonists, and probiotics/prebiotics as a means to prevent and combat NAFLD. The newest drugs pegozafermin and resmetiron, which seem to be very promising, arealso discussed.

## 1. Introduction

Non-alcoholic fatty liver disease (NAFLD) constitutes a spectrum of disorders ranging from merely hepatic steatosis, to the constellation of inflammation beyond liver steatosis, an entity known as non-alcoholic steatohepatitis (NASH) [1,2,3]. NAFLD may lead to chronic liver disease with various degrees of fibrosis and cirrhosis [1,2,3]. Finally, hepatocellular carcinoma may develop as a result of NAFLD [1,2,3]. Notably, NASH is considered the most rapidly rising cause of hepatocellular carcinoma worldwide [4,5]. In addition, NASH is the most frequent cause of liver transplantation among females and the second most common indication for liver transplantation among males in the United States of America [6,7,8].

NAFLD is characterized by fat accumulation in >5% of the hepatic cells, when other risk factors for lipid aggregation, such as alcohol or drugs, have been excluded [9]. NAFLD prevalence is on the rise, mainly due to the presence of the obesity epidemic. Nowadays, NAFLD has been suggested to affect approximately 25% of the population worldwide [1,2,3]. Moreover, its mortality is projected to approach an increase of 65% to 100% by 2030 in Asian-Pacific regions [10]. It is noteworthy that Riazzi et al., in their systematic review and meta-analysis reported an alarmingly increasing global prevalence of NAFLD before 2005, when compared to 2016 and later on [11]. They have found a greater increase among men than women regarding the prevalence of NAFLD in their meta-analysis [11].

Metabolic dysfunction associated with fatty liver disease or MAFLD, has been more recently than NAFLD been instituted as a definition to better describe the metabolic components of MAFLD. In particular, MAFLD is fatty liver disease associated with overweight/obesity and/or T2DM [12,13,14,15]. However, as MAFLD has only recently been added as a definition, there is still a lack of data regarding its exact prevalence. Therefore, in this review, we have chosen to refer to NAFLD instead of MAFLD.

As NAFLD poses a public health problem worldwide, we aimed to describe its pathogenetic mechanisms and diagnostic modalities, apart from liver biopsy. More specifically, serum biomarkers that could serve as a means to diagnose NAFLD, incorporated into various scores together with specific imaging methods, are further analyzed. Furthermore, monitoring of NAFLD in the clinical setting as well as potential therapeutic agents, that could be used and added to our armamentarium against NAFLD are elaborated upon.

## 2. NAFLD Pathogenesis

**Figure 1:**
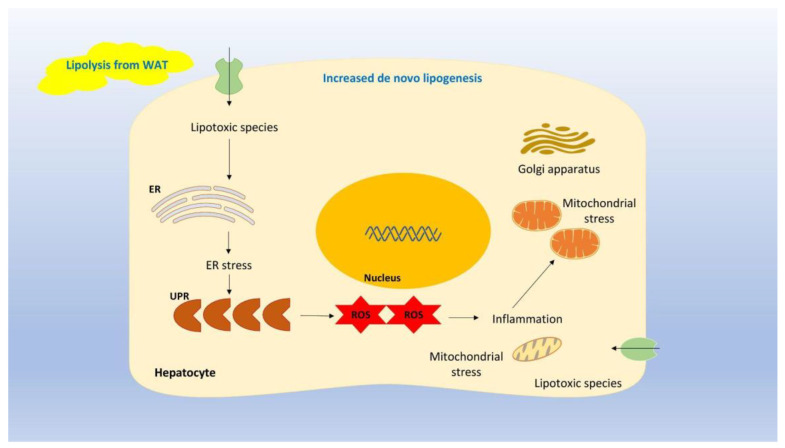
Legend: Excess fat accumulation in the liver may lead to the production of lipotoxic species, which induce ER stress and mitochondrial dysfunction. In particular, the ER is implicated in protein maturation. Excess lipid formation exceeds the ER’s capacity to produce mature proteins; thereby, unfolded proteins accumulate. This increase in unfolded proteins activates a cascade known as the UPR. Chronic ER stress may induce the UPR cascade, which, in turn, may lead to severe inflammation, the production of ROS, and ultimately, cell death.

NAFLD is synonymous with lipid accumulation in the liver, which is due to an imbalance between energy intake and energy consumption. Increased energy intake and/or decreased energy consumption leads to lipid storage apart from white adipose tissue (WAT) in ectopic areas, such as the liver, among others [16,17]. NAFLD results from de novo lipogenesis from carbohydrates in the liver as well as from lipolysis in WAT [1]. Free fatty acid efflux from WAT together with lipogenesis in the liver leads to hepatic fat aggregation. Excess fat accumulation in the liver may, in turn, lead to the production of lipotoxic species, which induce endoplasmic reticulum (ER) stress and mitochondrial dysfunction [1]. In particular, the ER is implicated in protein maturation. Excess lipid formation exceeds the ER‘s capacity to produce mature proteins; thereby, unfolded proteins accumulate. This increase in unfolded proteins activates a cascade widely known as the unfolded protein response (UPR). Despite the fact that UPR is activated to promote homeostasis, chronic ER stress may induce the UPR cascade, which, in turn, may lead to severe inflammation, the production of reactive oxygen species (ROS), and, finally, cell death [18,19,20,21,22,23,24,25]. Figure 1 depicts the involvement of UPR in ER stress and inflammation.

**Figure 2:**
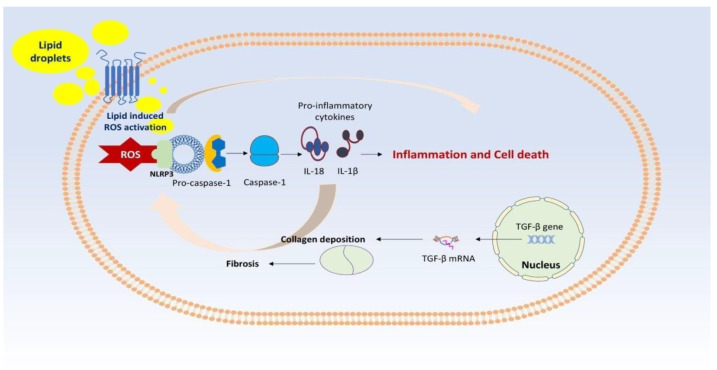
Legend: ROS binds to NLRP3, an inflammasome that, when activated by the canonical inflammasome pathway, i.e., via pro-caspase-1, results in the production of caspase-1. Caspase-1 leads to transformational alterations in NLRP3, which provoke the production of the pro-inflammatory cytokines IL-18 and IL-1β. This vicious cycle accounts for the inflammation seen in NASH. In addition, collagen deposition, which is mainly due to the activation of TGF-βresults in fibrosis seen in NAFLD.

In addition, ROS binds to nucleotide-binding domain-like receptor protein 3 (NLRP3), an inflammasome that, when activated by the canonical inflammasome pathway, i.e., via pro-caspase-1, results in the production of caspase-1. Caspase-1 leads to transformational alterations in NLRP3, which provoke the production of the pro-inflammatory cytokines IL-18 (interleukin-18) and IL-1β (interleukin-1β) [18,19,20,21,22,23,24,25]. This vicious cycle accounts for the inflammation seen in NASH. In addition, collagen deposition, which is mainly due to the activation of transforming growth factor beta (TGF-β) is responsible for the various degrees of fibrosis seen in NAFLD. Therefore, increased levels of TGF-β together with a long duration of NAFLD may ultimately result in cirrhosis [26]. Figure 2 depicts the implication of the inflammasome and TGF-β signaling regarding the severity of NAFLD.

**Figure 3:**
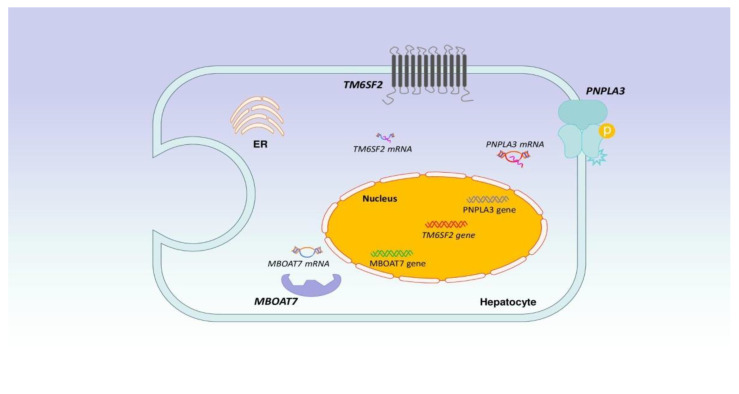
Legend: Literally, *PNPLA3*, *TM6SF2, GCKR* and *MBOAT7* are major genes associated with NAFLD. More specifically, GWAS have demonstrated that SNPs in the above-mentioned genes have been related to NAFLD development.

As previously mentioned, NAFLD seems to be the consequence of fat accumulation, inflammation, increased production of ROS, hepatic cell damage, and apoptosis. The “multi-hit” hypothesis refers to the interplay of genetic and environmental factors, such as diet, the gut microbiota, and hormonal components, with hepatic fat aggregation lying at the heart of the problem [27,28,29,30,31,32,33,34,35,36]. Literally, patatin-like phospholipase domain-containing protein 3(*PNPLA3*),atransmembrane 6 superfamily member 2 (*TM6SF2*), glucokinase regulatory protein (*GCKR*), and membrane-boundO-acyltransferase 7(*MBOAT7*)are major genes associated with NAFLD. More specifically, genome-wide association studies (GWAS) have demonstrated that single nucleotide polymorphisms (SNPs) in the above-mentioned genes have been related to NAFLD development [28]. SNPs in *PNPLA3* have been more extensively studied than SNPs in other genes. Notably, in a study involving the U.S. population, carriers of PNPLA3 I148M had higher liver fat and were characterized by increased mortality from liver disease and all-cause mortality [29]. As the SNP list is continuing to grow, with the latest being SIRT5 rs12216101 T>G among patients with NAFLD, there is much interest regarding the genetic and epigenetic factors involved [30]. Figure 3 depicts the main genes/components that are implicated in the development of NAFLD.

In terms of diet, the classical animal model of NAFLD is that of the methionine-choline-deficient (MCD) mouse model. In addition, a high-fat diet, a diet rich in fructose or sugar-containing beverages, together with a sedentary lifestyle, all contribute to the development of NAFLD [31,32,33,34,35,36,37]. Furthermore, the role of the gut microbiota is increasingly being recognized as crucial in this complex process. In particular, patients with NAFLD and especially with NASH have been documented to have lower concentrations of *Firmicutes* and higher levels of *Bacteroidetes*, thus a decreased *Firmicutes* to *Bacteroidetes* ratio (*F/B*). Despite the fact that not all studies agree with the decreased *F/B* ratio, this finding cannot be overlooked as it has been reported in many studies. Furthermore, F/B ratio estimation varies greatly as it is highly dependent on the molecular method used for the identification of the gut microbiota. For example, 16S rRNA sequencing may yield different results compared to shotgun metagenome sequencing. Therefore, the method used to describe the gut microbiota may account for the conflicting results regarding the *F/B* ratio. However, the F/B ratio, although decreased in NASH patients, has not been found to be decreased among patients with NAFLD and hepatocellular carcinoma [31,32,33,34,35,36,37].

## 3. NAFLD Monitoring in the Clinical Setting

Undoubtedly, liver biopsy is considered the major diagnostic method for determining NAFLD/NASH and the various degrees of cirrhosis [38]. However, liver biopsy, as an invasive method, has been associated with adverse side effects, the most dangerous being hemorrhage. Hemorrhage, which may be life-threatening, occurs at a rate of 0,6% to 1% [39]. In addition, there is always intra- and inter-reader variability, as the reported degree of fibrosis as well as hepatic cell ballooning may vary between different histopathologists [40]. Notably, Davison et al. have reported that this suboptimal evaluation in liver biopsies may affect the enrollment together with the outcomes of clinical trials [40]. To overcome these discrepancies, a NAFLD activity score (NAS) was developed by the NASH Clinical Research Network in 2005 [41]. NAS is a score that takes into account the levels of steatosis (0–3), inflammation (0–3), ballooning (0–2) and separately the degree of fibrosis (0–4) [41]. NAS has been widely adopted in landmark studies to evaluate the therapeutic potential of vitamin E and pioglitazone among patients with NASH [42,43,44]. Nevertheless, the variations between different histopathologists’ assessments together with the invasive nature of liver biopsy still remain a major problem. Therefore, there is a growing need for non-invasive techniques to assess the degree of inflammation and fibrosis in NAFLD/NASH. This assessment may have significant implications for the monitoring and management of these patients. Non-invasive techniques are increasingly being used for the evaluation of patients with NAFLD/NASH. These techniques may be classified as scores based on serum biomarkers and imaging modalities’ scores.

### 3.1. Scores Based upon Serum Biomarkers

Among the several existing scores regarding the severity of NAFLD/NASH, the Fibrosis-4 index (FIB-4) and the NAFLD fibrosis score (NFS) are the most widely used nowadays [45,46,47,48]. The FIB-4 score requires information such as age, aspartate transferase (AST), alanine transferase (ALT) and platelet count (PLT). FIB-4, when using a cut-off of <1.30, has a negative predictive value (NPV) that approaches 95% [45,46,47,48]. However, its accuracy is limited as it may vary among different populations and among individuals within different age groups [45,46,47,48]. In particular, it has been estimated that approximately 30% of the individuals studied are categorized within the indeterminate range. Nevertheless, it is a very helpful tool, which is easy to apply for the evaluation of patients with NAFLD/NASH and has been demonstrated to be associated with adverse effects among these patients. Regarding NFS, it requires informationsuch as age, body mass index (BMI), diabetes, AST, ALT, PLT, and albumin serum levels [49,50]. Torres et al. have recently documented that FIB-4 using a cut-off of 1.505, has a sensitivity and specificity of 85%, whereas when using the NFS with a cut-off of −0.835, its sensitivity approaches 100%, with 70% specificity [49].

Apart from the aforementioned scores, a tool that is particularly useful to assess patients within the indeterminate range with the FIB-4 score has been adopted. This tool is based on the turnover of extracellular matrix (ECM) substances. More specifically, this score, known as the enhanced liver fibrosis (ELF) score, is calculated by using the levels of hyaluronic acid, tissue inhibitor of metalloproteinase 1 (TIMP-1), and N-terminal procollagen peptide III (PIIINP) [51,52]. An ELF threshold of 9.8 has been related to advanced fibrosis and, thereby, has been determined to be a critical score for adverse outcomes among patients with NAFLD/NASH [51,52]. Furthermore, an ELF threshold of 10.51 has been reported to exhibit a sensitivity of 51% and a specificity of 93% for advanced fibrosis [51,52]. Furthermore, a significant score, known as NIS4, uses the combination of four biomarkers: miR-34a-5p, α2-macroglobulin, YKL-40, and glycated hemoglobin (HbA1c) [53]. The NIS4 has been developed to identify patients with “at-risk NASH”, i.e., patients with a NAS ≥ 4 and fibrosis ≥ 2 [54]. The determination of patients with “at-risk NASH” is of the utmost importance, as this category of patients with progressive NASH should be closely monitored and managed. The NIS4 has been demonstrated to perform well for the identification of patients with “at-risk NASH” [50,53].More specifically, a cut-off point of <0.36 has a sensitivity of 82% and an NPV of 77.9% for identifying patients with “at-risk NASH”, whereas a cut-off point of >0.63 has a reported specificity of 87% [50,53]. Very recently, an improved version of the NIS4 tool has been developed, the NIS2+^TM^, which uses miR-34a-5p and YKL-40 together with a sex-adjusted component(sex∗miR-34a-5p). This NIS2+^TM^ has been documented to better identify patients with MASH who could benefit from liver biopsy [55]. This score had a liver biopsy failure rate of 39% when using a cut-off value of 0.53, compared toa liver biopsy failure rate of 58% when using the FIB-4 score with an associated cut-off value of 0.58 [55]. In addition, Harrison et al. have already studied and validated the NIS2+^TM^ technology, compared to NIS4 for patients with “at-risk NASH” [56]. For patients with “at-risk NASH”, the fibrotic NASH index (FNI) has also been developed. FNI requires information such as AST, high-density lipoprotein (HDL), and HbA1c to further evaluate those patients. FNI has been validated in a cohort of 264 patients with BMI ≥40 kg/m^2^ in an Italian study, which found that when using a cut-off of 0.10, the NPV was 93% [57].

Overall, tools based on biomarkers are useful, but they have limitations regarding their accuracy. Therefore, scores based on imaging techniques have also been developed.

### 3.2. Scores Based upon Imaging Modalities

The two main scores based on imaging techniques to evaluate the degree of fibrosis in NAFLD/NASH are vibration-controlled transient elastography (VCTE), which is ultrasound-based, and magnetic resonance elastography (MRE). Both use liver stiffness as a means to assess liver fibrosis [50,58]. MRE is being increasingly used with the implementation of different protocols. Three-dimensional MRE (3D-MRE) using 40 Hz and a threshold of 2.43 kPa has been estimated to exhibit a sensitivity and an NPV of 100% for advanced fibrosis [59]. MRE has also been demonstrated to have a sensitivity of 86%, a specificity of 91%, and an NPV of 97%, when using a cut-off of 3.63 kPa for diagnosing fibrosis stages 3 or 4 [60]. In addition, magnetic resonance imaging-derived proton density fat fraction (MRI-PDFF) has been developed as a diagnostic method for liver steatosis characterized by high accuracy [1,61]. More specifically, a proton density fat fraction of >8–10% has been determined as an inclusion criterion for patients with NAFLD in most original studies [1,61]. A reduction in fat fraction of ≥30% has been appointed as a useful endpoint in many clinical trials [62,63]. Moreover, this ≥30% decrease in fat fraction on MRI-PDFF has been shown to be predictive of a one-degree reduction in liver fibrosis in a clinical trial conducted by Tamaki et al. Tamaki et al. documented that finding, confirming the utility of MRI-PDFF, by comparing this reduction in fat fraction with histological improvement of fibrosis among patients with NASH [63,64].

### 3.3. A Combination of Serum Biomarkers and Imaging Modalities

As the prevention of cirrhosis is of paramount importance among patients with NAFLD/NASH, hepatologists have endeavored to develop a better method to predict the occurrence of fibrotic NASH. For that purpose, they have attempted to combine serological biomarkers with imaging study parameters to achieve improved accuracy regarding fibrotic NASH. In that context, the MAST score, which combines MRI data with serum AST levels, has been developed. The MAST score relies on MRI-PDFF for the assessment of liver steatosis, MRE for fibrosis, and AST as a means of activity, and has been demonstrated to exhibit good accuracy [65,66]. Noureddin et al. have documented that MAST was superior in discerning patients with fibrotic NASH compared to FAST. FAST is another combination score based on FibroScan to evaluate liver fibrosis degree and AST to assess activity [65]. Furthermore, MAST has been shown to be an accurate tool inpredicting major adverse liver outcomes (MALOs), such as ascites, hepatic encephalopathy, bleeding varices, liver-associated deaths, and hepatocellular carcinoma among patients with NASH [66]. Another combination score is the MEFIB, which is based on MRE for fibrosis and the FIB-4 score (age, AST, ALT, PLT). The MEFIB has been found to outperform MAST and FAST in detecting significant fibrosis, i.e., ≥2 stage, and “at-risk NASH” patients in a study performed by Kim et al. [67]. It is noteworthy that a meta-analysis of six cohorts showed that when an MEFIB score of ≥3.3 kPa on MRE was combined with an FIB-4 ≥ 1.6, this combination could discriminate patients with hepatic decompensation within five years with an NPV of 99.1% [68].

It should be pointed out that MEFIB, MAST and FAST are very useful diagnostic and prognostic tools among patients with NASH. Moreover, we should bear in mind that they are not competitors, but helpful and supplementary tools in diagnosing and staging patients with NASH [69]. Overall, regarding steatosis scores, it should be noted that their incorporation into routine clinical practice is generally limited due to their diagnostic efficacy, such as variability in patient cohorts and validation against imaging procedures and liver biopsy [70]. There is a debate on their added diagnostic information compared to routinely performed laboratory and imaging studies in cases suspected of NAFLD. Moreover, data have shown underperformance of these scores in cases suffering from comorbidities, such as T2DM and obesity, further restricting their routine implementation [71]. Moreover, previously developed scores should undergo validation employing the newly defined criteria for NAFLD/MAFLD while recognizing the heterogeneity of the disorder by including a wider range of clinical and case profiles to guarantee real-world representation and combining more than one test and score, incorporating non-invasive imaging. In conclusion, the complementary use of serum biomarkers together with imaging modalities seems to offer additive value in this context.

## 4. Prevention of NAFLD

As hepatic fat accumulation lies at the heart of NAFLD development, inhibition of excess fat is of the utmost importance. Therefore, to prevent excess and ectopic fat deposition, individuals should try to achieve a negative energy balance. Reduction in food intake and increased energy expenditure remain the cornerstones of NAFLD’s prevention [72].

Dietary interventions play a crucial role in managing NAFLD. A diet that promotes weight loss and improves insulin sensitivity is often recommended. In a recent meta-analysis of various interventions in NAFLD, there was a dose-response association between the degree of calorie restriction and the favorable effects on weight loss and liver function, advocating that this approach remains the key component of NAFLD management [73]. Carbohydrate limitations during the early stages of weight loss may be beneficial; however, at further stages, the amount of weight loss preponderates over diet composition [74]. In contrast, during weight stability, limiting calories from fat may be advantageous for diminishing liver fat. The degree of dietary fat saturation and the carbohydrate glycemic index present inconsistent effects on intrahepatic triglyceride content. Interestingly, the matrix of certain foods, such as dairy, has been inversely related to NAFLD [74]. Meta-analyses of randomized control trials (RCTs) have shown that the Mediterranean diet may reduce indirect and direct outcomes associated with NAFLD severity, such as liver fibrosis, total cholesterol, waist circumference, and liver enzymes [75,76]. There is limited, albeit moderate- to high-quality, evidence from a meta-analysis that intermittent fasting may ameliorate hepatic endpoints, such as liver enzymes, hepatic steatosis and stiffness, and promote weight loss in patients with NAFLD [77].

Regarding nutrition, the Mediterranean diet and the Paleolithic diet have been demonstrated to exert beneficial effects [78,79,80]. The Mediterranean diet is rich in fruits and vegetables, allows moderate consumption of fish, and avoids red meat. The Paleolithic diet, also known as “the stone-age diet”, encourages the consumption of fruits, vegetables, and meat, whereas it excludes dairy products, sugar, and all processed foods. Notably, Otten et al. have recently demonstrated the effectiveness of the Paleolithic diet in reducing hepatic fat among obese patients with type 2 diabetes mellitus (T2DM) [80]. In addition, increased intake of polyphenols, such as resveratrol, as well as other substances, such as curcumin and silibinin, a flavonoid stemming from milk thistle, have been suggested to reduce hepatic fat accumulation. Apart from promoting *β*-oxidation in the liver, nutraceuticals also possess antioxidant and anti-inflammatory properties [13,81,82]. In this context, the role of probiotics, prebiotics and synbiotics remains controversial [33,34,35]. Based on a recent meta-analysis of RCTs, probiotics/prebiotics/synbiotics could ameliorate energy metabolism biomarkers such as insulin, HOMA-IR and lipid biomarkers in the NAFLD population; however, these actions should be confirmed by larger studies [83]. Table 1 describes various nutraceuticals in terms of the prevention or progression of NAFLD.

Apart from diet and nutrition, enhanced energy expenditure, i.e., regular exercise together with the avoidance of a sedentary lifestyle, should be equally pursued [92]. Physical exercise may significantly improve liver function in patients with NAFLD. In a recent network meta-analysis, the best modality of exercise for NAFLD cases is the combination of aerobic and resistance training, which presents different effects on various indicators of NAFLD, such as liver enzymes and lipid biomarkers [93]. In another meta-analysis of RCTs, exercise ameliorated indicators in patients with NAFLD and T2DM; however, the improvement in NAFLD indicators varied by the type of exercise (high-intensity interval training, moderate-intensity continuous training, or resistance training) [94]. The effect of exercise on NAFLD indicators is particularly increased when physical activity lasts longer than 3 months [95]. Nevertheless, it is important to mention that exercise alone could not induce significant histopathological changes in biopsy-proven NAFLD [96]. Finally, personalized approaches tailored to individual needs and preferences are essential for the successful management of NAFLD.

## 5. NAFLD Treatment Options

Lifestyle modification, such as abstinence from alcohol and weight loss of at least 5% of the entirebody weight, is of paramount importance. Diet, nutrition, and exercise together with the usual anti-obesity measures should be followed. Among patients with NASH, the weight loss goal should be further increased to 7% and 10% of body weight. Referral to a multidisciplinary team, including a nutritionist, may be warranted, especially for patients who do not achieve normalization of serum ALT. Under these circumstances, additional weight loss more than 10% could be aimed for. The use of GLP-1 receptor agonists or even bariatric surgery could be applied if the weight loss goals are not met during a six-month period of lifestyle interventions. Among patients with T2DM and NASH, pioglitazone and GLP-1 receptor analogues are advisable, whereas among patients with NASH but not T2DM, high-dose vitamin E has been demonstrated to decrease steatosis and inflammation as well [1]. Vitamin E at a dose of 800 IU daily has been associated with amelioration in steatosis and inflammation as well as a reduction in serum ALT levels. However, high doses of vitamin E have been alleged to be related to increased all-cause mortality in a minority of studies [97]. In addition, it is noteworthy that high doses of vitamin E have also been associated with an increased risk of bleeding, a point that should be of particular concern for patients with NAFLD and cirrhosis, while a plausible relationship between prostate cancer and high doses of vitamin E has been reported as well [97]. Nevertheless, the impact of confounding factors, such as the concurrent use of other nutraceuticals, could account for these discrepancies among various studies [1].

Notably, apart from the above-mentioned interventions, modifications of risk factors for CVD should be pursued. As patients with NAFLD/NASH are also at increased risk for CVD, monitoring them for CVD risks is essential for their proper management [92].

Besides, the addition of the 3-hydroxy-3-methylglutaryl coenzyme A reductase inhibitor atorvastatin and omega-3 fatty acids remains questionable, as there are conflicting results. As NAFLD/NASH poses a public health problem with growing incidence, there is ongoing research regarding its therapeutic approach. Indeed, farnesoid X receptor (FXR) agonists, such as obeticholic acid (OCA), are being explored in combination with atorvastatin in the CONTROL trial [98]. Obeticholic acid 25 mg alone has been documented to exert an antifibrotic effect among 931 patients with histologically proven NASH in the REGENERATE trial [99,100]. OCA 25 mg daily was generally well tolerated for four years of study duration and its long administration. The most prominent adverse effect was pruritus, whereas hyperlipidemia was also of concern, which was noted as well with a relative risk (RR) of 2.69 (95% CI: 1.85–3.92, *p* < 0.01) [99,100].

In addition, other drugs are under investigation for NASH, among which pegozafermin and resmetiron seem to be very promising [101,102,103,104,105]. Pegozafermin is a fibroblast growth factor 21 (FGF-21) analog with long-acting properties due to the presence of glyco-pegylation, i.e., pegylation at a specific site with glycosyltransferases [101]. When compared to native FGF-21, it has a much longer half-life, which allows for a once-every-two-weeks administration of the recombinant form of FGF-21 [101]. Pegozafermin has been developed for the treatment of NASH as well as for managing severe hypertriglyceridemia [101,102,103]. It has been suggested to exert pleiotropic functions, which are mainly mediated via adiponectin, resulting in a reduction in inflammation and fibrosis. Loomba et al. enrolled 219 patients with biopsy-proven NASH and fibrosis stage 2 or 3 in their phase 2b RCT. Patients were assigned to receive pegozafermin 15 mg or 30 mg once weekly or 44 mg once every two weeks subcutaneously or placebo [103]. Loomba et al. reported an improvement in fibrosis, which supported its continuation in a phase 3 study [103]. The most frequent adverse effects of pegozafermin were nausea and diarrhea [103].

Regarding resmetiron, it is an oral inhibitor of thyroid hormone receptor β (THR-β). THR-β has been documented to be implicated in liver activity, and thyroid hormone function has been shown to be reduced in cases of NASH [104]. Only recently, in 2024, a study of resmetiron as a once-daily administration a dose of 80 mg to 100 mg in a phase 3 trial among 966 patients with NASH was published. In this study, 322 patients received 80 mg of resmetiron, 323 received 100 mg of resmetiron and 321 received placebo. In this MAESTRO-NASH trial, Harrison et al. reported that both doses were better than placebo, as they improved NASH by at least one stage. Resmetiron was generally well tolerated, with diarrhea and nausea being the most frequent adverse effects [105]. Resmetiron has just gained accelerated FDA approval for the treatment of NASH with moderate to severe fibrosis on 14th March 2024.

Apart from pegozafermin and resmetiron, ZSP1601 is an investigational drug belonging to a first-in-class pan-phosphodiesterase (pan-PDE) inhibitor, which has been developed for the treatment of NASH [106]. This pan-PDE inhibitor has been suggested to decrease levels of TNF-α via its action on PDE-2, thus reducing inflammation in NASH [84]. Hu et al., in their phase 1b–2a RCT, enrolled 36 patients with NAFLD who received ZSP1601 50 mg once daily, 50 mg twice daily, 100 mg twice daily, or placebo. They concluded that ZSP1601, when administered for 28 days, resulted in amelioration of fibrosis, hepatic fat, and serum liver indices [106]. Adverse effects were mild, usually diarrhea, headache, and transient elevations in serum creatinine concentrations [106].

Overall, there is ongoing research regarding the management of NASH. However, this research is very demanding on account of the increasing incidence of NAFLD/NASH as well as inherent difficulties. First, the need for biopsy-proven NAFLD/NASH and the necessity for assessment by three expert pathologists in the field is one obstacle. Second, the determination of primary and secondary endpoints is also a significant issue. Third, the total duration of treatment in the relevant studies remains a major problem to be resolved. Fourth, the duration of total treatment required throughout a lifetime has not been answered so far [1].

## 6. Current Concepts and Future Perspectives

As NAFLD/NASH has been suggested to be multifactorial, its monitoring and management should be individualized [107,108,109,110,111,112,113,114,115,116]. Patients with different phenotypes of NAFLD/NASH should receive more tailored treatment based on genetics and epigenetics [107,108,109,110,111,112,113,114,115,116]. The heterogeneity of NAFLD/MAFLDhighlights the exciting potential of integrating multi-omics approaches (genetic variants as well as epigenetic, proteomics, metabolomics, lipidomics, and microbiomics biomarkers) as the future of non-invasive biomarkers, providing the prospect of personalized insights [108,109,110]. The confounding effects of age, gender, diet, medication, hormonal, and lifestyle parameters on these biomarkers (e.g., gut microbiota) must also be considered [108,109,110]. Nevertheless, to establish robust omics signatures, reported biomarkers must undergo validation in larger and more diverse patient cohorts. The validation process is important for improving our capacity to effectively transition from biomarker discovery to dealing with the challenges of diagnostic accuracy, cost-effectiveness, applicability, reproducibility, and feasibility in clinical practice.

Genetic scores have already been developed to evaluate the effectiveness of pioglitazone and obeticholic acid among patients with NAFLD/NASH [108,109]. As already aforementioned, the presence of the SNP PNPLA3 I148M has been demonstrated to be associated with increased liver and all-cause mortality. Moreover, patients with NASH and PNPLA3 I148M are more prone to present with a worse clinical condition but have been more responsive to treatment with lifestyle modifications and bariatric surgery. In contrast, the administration of omega-3 fatty acids in this category of patients is less efficacious [1]. This is just a simple paradigm for the need for a more personalized approach to the management of NAFLD/NASH. Despite the fact that clustering of NAFLD sub-phenotypes has recently been suggested based on several parameters, such as age, sex, BMI, diabetes, dyslipidemia, etc., it is likely that personalized management would be more rewarding and beneficial [107,108,109,110,111,112,113,114,115,116]. In this setting, the contribution of artificial intelligence (AI) is highly appreciated [1]. AI may be very helpful in addressing all these particular issues that emerge regarding the monitoring and management of patients with NAFLD/NASH [1]. It is noteworthy that, apart from the potential usefulness of AI, resmetiron has already gained FDA approval. In addition, the development of acidifying nanoparticles, which act at the lysosome level, seems to be a very promising approach [116].

## 7. Conclusions

As NAFLD/NASH represents a public health problem worldwide, the need for more effective markers based on a combination of serum biomarkers and imaging modalities for monitoring patients is mandatory. Various scores, such as NAS, NIS4, FAST, MAST, and MEFIB, may be used for assessing disease progression and should be regarded as additive values and not as competitive information. In terms of treatment options, pegozafermin and resmetiron seem to be promising therapeutic agents. Nevertheless, whether combination treatment with several drugs is beneficial or not should also be studied. As the pathogenesis of NAFLD/NASH is multifactorial, patients should be monitored and treated in a personalized manner based on genetic background and factors such as age, sex, BMI, and the presence or absence of other comorbidities. In this context, the contribution of AI together with a multidisciplinary team that would address each and every health issue of the patient would be more than welcome.

## Figures and Tables

**Table 1:** Main nutraceuticals and their quality of evidence in the prevention or progression of NAFLD.

Substance/Food	Recommendation/ Daily Dose	Mechanism of Action	Remarks
Artichoke Leaf Extract [81]	Very Low 1000–2000 mg	Lipid-lowering activity, mechanism largely unknown, possible via inhibition of HMG-CoA reductase	↓ LDL ↓ TGs ↓ AST and ALT AEs: very rare (flatulence)
Astaxanthin [82]	Very Low 5–20 mg	Mechanisms largely unknown Amelioration in liver fibrosis and inflammation	Generally safe. Further studies in humans are needed.
Berberine, can be found in roots and plants, especially Berberis genus [84]	Moderate 500–1500 mg	Antioxidant effects, effect on FXR and NF-κB, Activation of LDLRs, Inhibition of PCSK9	↓ liver steatosis ↓ IR ↓ LDL AEs: mild gastrointestinal (diarrhea, flatulence) Its poor bioavailability should be improved.
Catechins in Green Tea [85,86]	Low 250–1200 mg green tea extract	Inhibition of NF-κB, ↑ Apoptosis	Conflicting results regarding its efficacy, which are attributed to the antioxidant state of each individual.
Coenzyme Q10 [81,82]	Low 100–300 mg	↑ PPARs alpha and gamma, ↑ AMPK, anti-inflammatory and antioxidant role	↓ severity of NAFLD, ↓ GGT, AST and ALT. AEs: Conflicting results on muscle function.
Curcumin from Curcuma longa [81,82,87]	Low 100–1500 mg	Mechanism of action largely unknown, anti-inflammatory effects	↓ hepatic steatosis, ↓ AST. AEs: mild, such as diarrhea, headache and rash.
Omega 3 Fatty Acids [81,82]	Moderate 2–4 g EPA and DHA	↓ VLDL, ↓ enzymes for the synthesis of TG, ↑ beta oxidation of fatty acids, ↓ proinflammatory cytokines, such as IL-6 and TNF-α, ↓ activation of NF-κB, ↓ IR	↓ hepatic steatosis ↓ TGs. AEs: mild gastrointestinal
Prebiotics, such as oligofructose, inulin and psyllium [81,82]	Very Low	↓ IR ↓ Inflammation via the modulation of gut microbiota	↓ liver steatosis Generally safe.
Probiotics, such as *Lactobacillus* and *Bifidobacteria* and next-generation probiotics, such as *Akkermansiamuciniphila* and *Faecalibacteriumprausnitzii* [88,89]	Very Low 1–100 billion	↓ IR ↓ Inflammation via the modulation of gut microbiota	↓ liver steatosis Generally safe.
Resveratrol [81,82,87]	Very Low 100–300 mg	Activation of AMPK/SIRT1 axis, ↓ ROS, Induces Autophagy	Conflicting results regarding its efficacy. AEs: if dose >2500 mg
Silymarin from milk thistle [90,91]	Moderate 100–500 mg	Scavenger of ROS, ↓ activation of NF-κB, ↓ Procollagen III and TGF-β, ↑ PPAR action	↓ liver steatosis and fibrosis AEs: non-significant Its poor bioavailability must be improved.
Vitamin D [81]	Very Low 1000–5000 UI	Immuno-modulatory and anti-inflammatory effects, effect on TLRs, which are implicated in the pathogenesis of NAFLD	Generally safe.
Vitamin E [81]	Very Low 100–1200 UI	Scavenger of ROS	Conflicting results about its safety; should be further investigated at high doses.

Abbreviations: AEs: Adverse Effects; AMPK: AMP-activated Protein Kinase; ALT: Alanine-Transferase; AST: Aspartate-Transferase;GGT: Gamma Glutamyl Transferase; HMG-CoA: Hydroxy-Methyl-Glutaryl Coenzyme A; IL-6: Interleukin-6; IR: Insulin Resistance; LDL: Low Density Lipoprotein; NAFLD: Non Alcoholic Fatty Liver Disease; NF-κB: Nuclear Factor kappa B; PCSK9:Pro-protein Convertase Subtilisin/Kexin type 9; ROS: Reactive Oxygen Species; SIRT1: Sirtuin 1; TGs: Triglycerides; TGF-β: Tumor Growth Factor beta; TLRs: Toll-like Receptors; TNF-a: Tumor Necrosis Factor alpha; VLDL: Very Low Density Lipoprotein.
